# The Role of Bicarbonate Therapy in Diabetic Ketoacidosis: A Systematic Review and Meta‐Analysis

**DOI:** 10.1002/edm2.70191

**Published:** 2026-03-09

**Authors:** Abu Omayer, Anil KC, Arsalan Sharif, Syed Mohammed Hassanul Hoque, Hasan BaniHani, Lana Khaled, Waseem Sajjad, Moussa Nassar, Ahmad Holeihel

**Affiliations:** ^1^ Mohammed Bin Rashid University of Medicine and Health Sciences Dubai UAE; ^2^ Mayo Clinic Rochester Minnesota USA; ^3^ Fakeeh University Hospital Dubai UAE; ^4^ University of Georgia Tbilisi Georgia; ^5^ Department of Neurologic Surgery, Mayo Clinic Rochester Minnesota USA; ^6^ University of Sharjah Sharjah UAE; ^7^ King Edward Medical University, Mayo Hospital Lahore Pakistan; ^8^ Lebanese American University School of Medicine Byblos Lebanon; ^9^ University Hospital of Sharjah Sharjah UAE

**Keywords:** bicarbonate therapy, diabetes mellitus, diabetic ketoacidosis, DKA

## Abstract

**Background:**

Diabetic ketoacidosis (DKA) is a severe diabetes complication managed with fluids, insulin and electrolyte correction. This review evaluates the debated effectiveness of bicarbonate therapy on DKA outcomes.

**Methods:**

Following PRISMA guidelines, we systematically reviewed studies on bicarbonate therapy in DKA. We searched PubMed, Google Scholar, the Cochrane Library and ClinicalTrials.gov (as of August 2024), including studies on patients with DKA. Meta‐analysis was conducted using RevMan. Bias risk was assessed with the Newcastle‐Ottawa Scale (NOS) for cohort studies, Cochrane's ROB‐2 for RCTs and the Joanna Briggs Institute (JBI) Scale for case series. Prospero ID: CRD42024581810.

**Results:**

Eight studies, including 646 patients, met the inclusion criteria. Participants' mean ages spanned from approximately 9.7 years to 45.8 years. Bicarbonate therapy did not significantly improve pH levels (mean difference = −0.02, 95% CI [−0.13, 0.09], *p* = 0.7), time to resolution of acidosis (mean difference = 0.09 h, 95% CI [−2.6, 2.79], *p* = 0.95) or potassium levels (mean difference = −0.10, 95% CI [−0.49, 0.29], *p* = 0.61). Bicarbonate therapy was associated with a marginally longer duration of hospital stay (mean difference = 13.63 h, 95% CI [0.23, 27.03], *p* = 0.05), although the overall effect size was small. No significant difference was observed in the incidence of hypoglycemia (odds ratio = 2.62, 95% CI [0.59, 11.63], *p* = 0.20). High heterogeneity was observed across most outcomes, mainly due to variability in study protocols and patient populations.

**Conclusion:**

Bicarbonate therapy provided no meaningful clinical benefit in the routine management of DKA and was associated with potential harm, including worsened hyperglycemia. Consistent with current guidelines, its use should be restricted to cases of severe acidemia (pH < 6.9). Further high‐quality studies are needed to clarify its role in extreme acidosis and to explore alternative buffering strategies.

## Graphical abstract

This meta‐analysis of 646 patients shows that bicarbonate therapy does not improve acidosis resolution, pH or electrolyte outcomes in diabetic ketoacidosis and may prolong hospitalisation and worsen hyperglycemia. Routine use is unsupported and should be limited to severe acidemia (pH < 6.9).

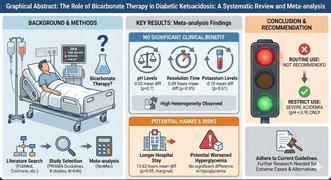

## 1 Introduction

Diabetic ketoacidosis (DKA) is a serious, potentially life‐threatening acute complication of diabetes mellitus, characterised by hyperglycemia (blood glucose > 250 mg/dL), metabolic acidosis (pH < 7.3, serum bicarbonate < 18 mEq/L) and ketonemia [1, 2]. It occurs in both type 1 and type 2 diabetes and remains a major cause of diabetes‐related morbidity and mortality worldwide. Mortality rates range from 2% to 5% in industrialised settings but rise to 6%–24% in low‐resource regions [3]. DKA also carries a substantial clinical burden, contributing to frequent hospitalizations and serious complications, including cerebral edema, particularly in children [4, 5].

Optimal DKA management involves restoring intravascular volume, correcting electrolyte abnormalities and resolving ketosis. Current ADA and ISPAD guidelines recommend initiating resuscitation with 0.9% saline, followed by individualised adjustment of fluid type and rate based on hemodynamic status and corrected sodium concentration. Patients with normal or elevated corrected sodium may transition to 0.45% saline, whereas those with low corrected sodium typically continue 0.9% saline. Continuous electrolyte monitoring guides subsequent adjustments. Insulin therapy is central to reversing the metabolic derangements of DKA, with continuous intravenous insulin infusion remaining the standard of care—particularly in paediatric patients—while subcutaneous rapid‐acting analogs may be considered only in select adults with mild to moderate DKA. Electrolyte replacement—especially potassium—is essential, as DKA is associated with marked total body potassium deficits. Insulin should be withheld in cases of severe hypokalemia until potassium levels are adequately corrected, and routine phosphate or bicarbonate supplementation is not recommended except when clinically indicated [5, 6].

Bicarbonate therapy has historically been considered for severe metabolic acidosis in DKA, particularly when arterial pH falls below 6.9. However, its use remains controversial. The American Diabetes Association (ADA) discourages routine bicarbonate administration due to limited evidence of benefit and the potential for harm [7]. Some studies report transient improvements in acidemia without meaningful clinical benefit (systematic reviews and trials). Guidelines differ on whether to consider bicarbonate for very severe acidosis (e.g., venous/arterial pH < 6.9), but randomised and cohort data have not demonstrated consistent clinical benefits even in patients with pH < 7.0 [7, 8, 9, 10]. Major concerns include delayed ketone clearance, hypokalemia and the possibility of worsening acidosis at the cellular level [7, 8, 10].

The physiological actions of bicarbonate help contextualise this debate. Bicarbonate buffers hydrogen ions to form carbonic acid, which dissociates into water and carbon dioxide [11, 12]. While this reaction raises extracellular pH, the generated CO_2_ diffuses across cell membranes more rapidly than bicarbonate, potentially causing paradoxical intracellular acidosis [13, 14, 15]. This mechanism has been implicated in neurologic complications, including cerebral edema, particularly in paediatric DKA [16]. Additional factors—such as hyperchloremia during resuscitation, which can artificially lower measured bicarbonate and base deficit—may further complicate acid–base interpretation and influence decisions about bicarbonate use [17]. Conversely, although early correction of acidosis has been theorised to improve insulin responsiveness [18, 19], no clinical evidence supports a meaningful benefit of bicarbonate therapy in this context.

Despite decades of clinical experience, the efficacy and safety of bicarbonate therapy in DKA remain uncertain and recommendations continue to vary. Importantly, no previous systematic review has quantitatively synthesised available data to clarify its impact on biochemical parameters or clinical outcomes.

The objective of this systematic review and meta‐analysis is therefore to evaluate the effects of bicarbonate therapy on key outcomes in DKA, including changes in pH, time to resolution of acidosis, hospital length of stay, potassium levels and the incidence of hypoglycemia. By addressing existing gaps in evidence, this study aims to inform clinical practice and support more precise guideline recommendations for the management of DKA.

## 2 Materials and Methods

The protocol for this systematic review and meta‐analysis was registered on PROSPERO using the following ID CRD42024581810, and our methodology follows the Preferred Reporting Items for Systematic Reviews and Meta‐Analyses (PRISMA) guidelines [20]. The PRISMA 2020 reporting checklist with manuscript page references is found in Table S1.

### 2.1 Search Strategy

We searched PubMed, Google Scholar, Cochrane Library and ClinicalTrials.gov from inception to 08 August 2024. To identify relevant studies, we used keywords such as *diabetic ketoacidosis*, *diabetic acidosis*, *bicarbonate* and *alkalinizing agents*. We also manually examined the bibliographies of each included article to identify additional studies that satisfied the inclusion criteria.

The full search strategy is linked in File S1:

(“diabetic ketoacidosis” OR “Diabetic Ketoacidosis”[Mesh] OR “diabetic ketosis” OR DKA OR “diabetic acidosis” OR ketoacidosis) AND (bicarbonate OR “Bicarbonates”[Mesh] OR “sodium bicarbonate” OR “Buffering Agents”[Mesh] OR “hydrogen carbonates” OR “sodium hydrogen carbonate” OR “bicarbonate therapy” OR “alkalinizing agents”) AND (treatment OR treatments OR “Therapeutics”[Mesh] OR management OR intervention) AND (“Randomized Controlled Trial”[Publication Type] OR “Randomized Controlled Trials as Topic”[Mesh] OR “Clinical Trial”[Publication Type] OR “Clinical Trials as Topic”[Mesh] OR “Prospective Studies”[Mesh] OR “Cohort Studies”[Mesh] OR “Cross‐Sectional Studies”[Mesh] OR “Case Series”[Publication Type] OR “observational study” OR “retrospective study” OR “comparative study”).

### 2.2 Study Selection

For this meta‐analysis, studies were included if they evaluated patients of any age diagnosed with diabetic ketoacidosis (DKA), defined by hyperglycemia (plasma glucose greater than 250 mg/dL), metabolic acidosis (arterial pH ≤ 7.30 or serum bicarbonate < 15 mEq/L) and evidence of ketosis. Both paediatric and adult populations were considered, provided that the intervention included administration of intravenous sodium bicarbonate and a control group without bicarbonate therapy was available for comparison. Eligible studies reported clinical outcomes such as time to resolution of acidosis, length of hospital stay, etc. Studies were excluded if they involved patients with arterial pH below 6.9 or above 7.30. The rationale for selecting a pH range of 6.9 to 7.30 is grounded in both ethical and clinical considerations. International guidelines recommend the administration of bicarbonate for patients with a pH below 6.9 due to the severity of acidosis, making it ethically inappropriate to withhold treatment in this subgroup; as such, these patients cannot ethically contribute to a true no‐bicarbonate comparison group [21, 22].

Conversely, in patients with a pH above 7.30, the acidosis associated with DKA is considered mild or resolving, and current guidelines explicitly discourage the use of bicarbonate therapy in this setting [23, 24]. Therefore, this pH window represents the most clinically relevant and ethically permissible range for evaluating the comparative effectiveness of bicarbonate therapy.

Additional exclusion criteria included pregnancy and cases where DKA was a secondary diagnosis to a more critical illness.

Eligible study designs included randomised controlled trials, non‐randomised controlled trials, cohort studies (prospective or retrospective), case–control studies, cross‐sectional studies and case series. No restrictions on the year of publication or language were applied. Animal studies, reviews, editorials, commentaries, meta‐analyses and research articles without data on bicarbonate therapy were excluded. Studies without a comparison group were excluded as only a double‐armed meta‐analysis was performed (details in the Data Analysis section).

The Rayyan platform was employed for the screening process [25]. Study selection was performed in two steps: Title and abstract screening followed by full‐text screening. Three independent reviewers screened the articles according to the pre‐defined criteria, and a fourth reviewer was consulted to resolve any disputes. This protocol was followed during both the title and abstract screening and the full‐text screening.

### 2.3 Data Extraction, Quality Assessment and Ethical Aspects

Three independent reviewers extracted data from each study, followed by a comparison with the full text to ensure accuracy. A fourth reviewer resolved discrepancies when a consensus was not reached.

The following baseline and summary data were extracted from eligible studies: last name of the first author, year of publication, study design, sample size, country of study, mean age at examination (with standard deviation), percentage of males, baseline BMI and initial bicarbonate level. Outcomes of interest included duration of hospital stay, pH level, duration of acidosis or time to resolution of acidosis, potassium (K+) levels, glucose levels and HCO_3_ levels. These outcomes were compared between the bicarbonate therapy group and the control group.

### 2.4 Quality Assessment

- Cohort Studies: The risk of bias was assessed using the Newcastle–Ottawa Scale (NOS), which has a maximum score of nine points. Observational studies with an NOS score ≥ 7 were classified as high quality, while those scoring < 7 were considered low quality. Studies were categorised as follows: Very Good (9 points), Good (7–8 points), Satisfactory (5–6 points) and Unsatisfactory (0–4 points) [26].
- RCTs: Risk of bias for randomised controlled trials was assessed using Cochrane's ROB‐2 tool which is based on 5 domains: [1] bias arising from the randomization process, [2] bias due to deviations from intended interventions, [3] bias due to missing outcome data, [4] bias in the measurement of the outcome and [5] bias in the selection of the reported result.
- Case Series: The Joanna Briggs Institute (JBI) Scale was employed for quality assessment [27].

This systematic review is based solely on secondary data from published studies. As it does not involve human participants, ethical approval was not required.

### 2.5 Data Analysis

The meta‐analysis was performed using RevMan software, version 5.425. Only double‐armed meta‐analyses were conducted to compare outcomes between bicarbonate therapy and control groups. For continuous outcomes, mean difference (MD) with 95% CI was used because all studies reported outcomes on the same measurement scale; where units differed, they were converted to a common unit to ensure comparability and maintain clinical interpretability. Subgroup analyses were conducted for pH at 2 h. Sensitivity analyses were performed to identify heterogeneity sources using a leave‐one‐out approach. A fixed‐effect model was applied if heterogeneity was insignificant (*I* ^2^ < 50%), whereas a random‐effects model was used when significant heterogeneity was detected. Statistical significance was defined as *p* < 0.05.

### 2.6 Publication Bias Assessment

Publication bias was assessed through visual inspection of funnel plots for outcomes with more than four included studies. Although formal guidance recommends a minimum of ten studies for reliable interpretation of funnel plot asymmetry or statistical tests such as Egger's regression, funnel plots were generated to enhance transparency and meet reporting standards [28]. Due to the small number of studies available for each outcome, formal statistical tests for funnel plot asymmetry were not performed, and the results should be interpreted with caution.

## 3 Results

### 3.1 Literature Search and Screening

Application of our search strategy in PubMed, Cochrane, Google Scholar and ClinicalTrials.gov yielded a total of 1302 records. After removing duplicates (105 using Rayyan and 10 manually), 1187 records remained and underwent title and abstract screening. From these, 24 studies were sought for retrieval for full‐text screening.

**FIGURE 1:**
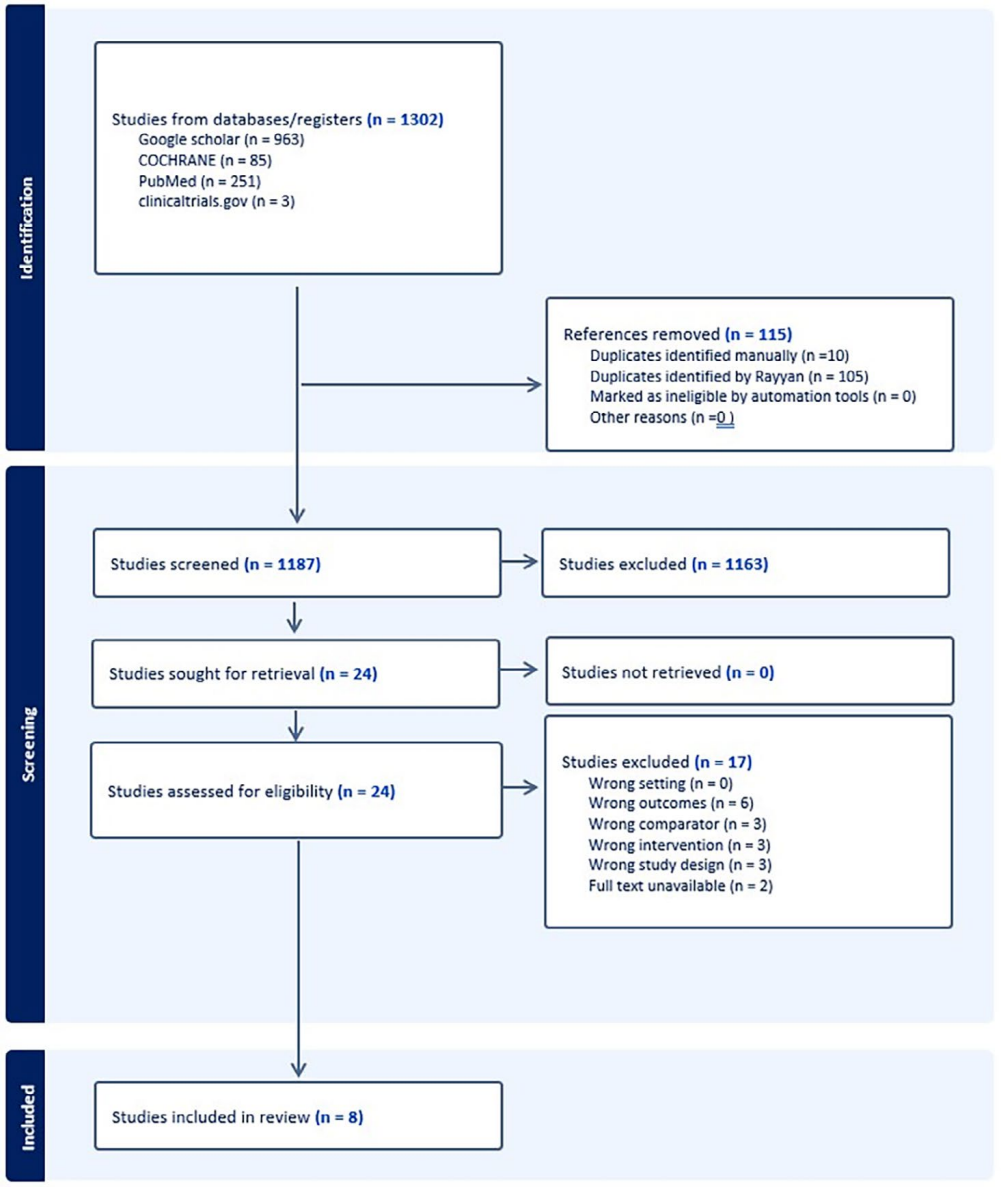
PRISMA 2020 flow diagram illustrating study identification, screening, eligibility assessment and final inclusion.

Following the screening process, we identified 8 studies for inclusion [10, 29, 30, 31, 32, 33, 34, 35]. This is detailed in the PRISMA flowchart in Figure 1.

The included studies represented diverse populations, with sample sizes ranging from 20 to 232 participants. These studies were conducted in various countries, including the USA, Turkey, the Netherlands, Mexico and France. Participants' mean ages spanned from approximately 9.7 years to 45.8 years. The proportion of male participants varied significantly, from 40.6% to 54.3%.

Initial pH values ranged from 6.89 to 7.05, reflecting the varied severity of the conditions under study. Baseline bicarbonate levels were reported in multiple studies, ranging from 2.66 mmol/L to 5.3 mmol/L. Potassium levels (K+) showed a wide spectrum from 3.92 mmol/L to 5.346 mmol/L. Baseline glucose levels were diverse, with values from 405.6 mg/dL to 763.7 mg/dL across studies. Further details are provided in Table 1.

**TABLE 1:** Baseline characteristics of included studies.

Study ID	Country	Study design	Initial pH		Sample size	Male%	AGE M (SD)	Initial Bicarbonate level (mmol/L), M (SD)	Bicarbonate dose given (mmol/L)	Timing of dose administration (How long after patient's arrival for DKA treatment was bicarbonate therapy given)	Initial K+ level (meq/L)	Initial glucose level (mg/dL)
			Bicarbonate M (SD)	Control M (SD)								
Ozturk 2023	Turkey	Retrospective cohort	6.98 ± 0.7	7.14 ± 0.11	232	54.3	9.7 ± 5.77	NA	NA	Within 2 h	4.1379 ± 0.7845 mmol/L	NA
Morris 1986	USA	RCT	7.2 ± 0.04	7.27 ± 0.03	21	NA	30.86 ± 5.36	3.36 ± 0.55	89.2 meq	NA	5.2838 ± 0.463 meq/L	508.143 ± 63.576 mg/dL
Marr 1981	USA	Retrospective cohort	NA	NA	131	40.6	10.30 ± 4.59	NA	NA	NA	4.845 ± 0.73 meq/L	498.357 ± 192.315 mg/dL
Lutterman 1979	Netherlands	Prospective cohort	6.99 ± 0.09	6.98 ± 0.08	24	NA	37.5 ± 15.1	NA	167 mmol	3 h	5.3 ± 1.7122 mmol/L	763.7 ± 271.561 mg/dL
Lever 1983	USA	Retrospective cohort	6.97 ± 0.07	6.98 ± 0.12	73	42.19	31.76 (SD not given)	3.92 ± 1.17	130 mmol	NA	NA	704.095 ± 293.845 mg/dL
Green 1998	USA	Case Series	7.02 ± 0.08	7.06 ± 0.08	106	42.69	9.83 ± 4.35	NA	NA	NA	5 ± 1.0298 meq/L	592.283 ± 168.631 mg/dL
Gamba 1991	Mexico	RCT	7.05 ± 0.08	7.042 ± 0.08	20	NA	28.45 ± 4.29	2.66 ± 0.98	NA	NA	4.52 ± 0.764 meq/L	405.6 ± 211.431 mg/dL
Viallon 1999	France	Retrospective cohort	6.98 ± 0.8	7.0 ± 0.08	39	53.6	45.77 ± 16.01	3.46 ± 6.70	NA	NA	5.346 ± 1.0035 mmol/L	697.254 ± 222.237 mg/dL

### 3.2 Risk of Bias Assessment

The risk of bias for five cohort studies was assessed using the Newcastle‐Ottawa Scale (NOS). Three studies scored 8, one scored 7 and one achieved a score of 9. Based on the scoring, one study was categorised as very good, three as good and one as satisfactory (Table 2).

**TABLE 2:** Newcastle‐Ottawa Scale for cohorts.

Author (Year)	Selection				Comparability	Outcomes			Total
	(1) Representativeness of the exposed cohort	(2) Selection of the non exposed cohort	(3) Ascertainment of exposure	(4) Demonstration that outcome of interest was not present at start of study	(1) Comparability of cohorts on the basis of the design or analysis	(1) Assessment of outcome	(2) Was follow‐up long enough for outcomes to occur	(3) Adequacy of follow up of cohorts	
Ozturk 2023	1	1	1	1	1	1	1	1	8
Marr 1981	1	1	1	1	1	1	1	1	8
Lutterman 1979	1	1	1	1	1	1	1	1	8
Lever 1983	1	1	1	1	0	1	1	1	7
Viallon 1999	1	1	1	1	2	1	1	1	9

**FIGURE 2:**
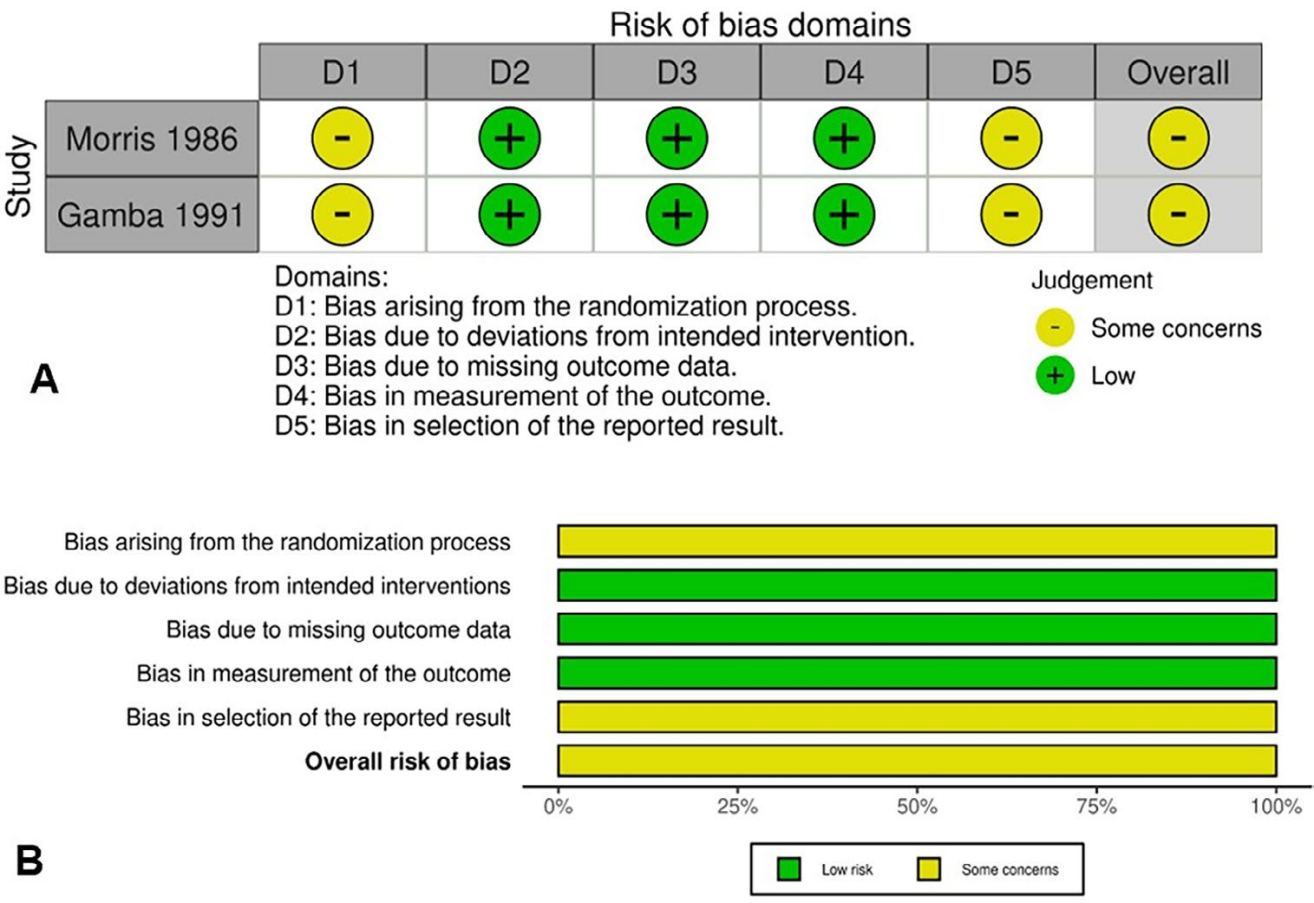
Risk of bias assessment for randomised controlled trials using the Cochrane Risk of Bias 2 tool: (A) domain‐level judgements per study; (B) summary of risk of bias across domains.

Two randomised controlled trials were assessed using the Cochrane RoB 2 tool. Both studies were rated as having some concerns due to issues with randomization processes and the selection of reported results (Figure 2).

One case series was assessed using the JBI critical appraisal tool, as given in Table 3. The study met most criteria but was rated ‘No’ for two items: complete inclusion of participants and clear reporting of the presenting site(s)/clinic(s) demographic information. Despite these limitations, the study was otherwise methodologically sound.

**TABLE 3:** JBI critical appraisal tool.

	Were there clear criteria for inclusion in the case series?	Was the condition measured in a standard, reliable way for all participants included in the case series?	Were valid methods used for identification of the condition for all participants included in the case series?	Did the case series have consecutive inclusion of participants?	Did the case series have complete inclusion of participants?	Was there clear reporting of the demographics of the participants in the study?	Was there clear reporting of clinical information of the participants?	Were the outcomes or follow up results of cases clearly reported?	Was there clear reporting of the presenting site(s)/clinic(s) demographic information?	Was statistical analysis appropriate?
Green 1998	Yes	Yes	Yes	Yes	No	Yes	Yes	Yes	No	Yes

### 3.3 pH Levels

**FIGURE 3:**
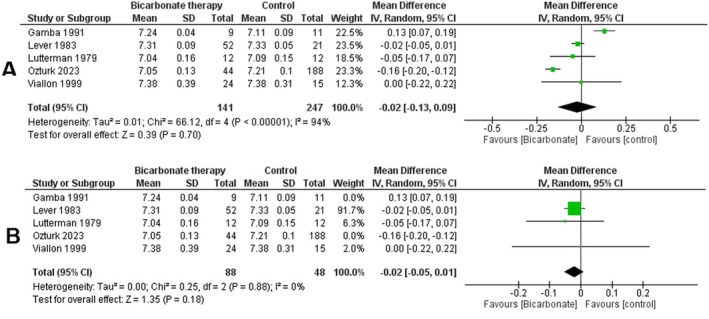
Forest plots comparing bicarbonate therapy versus control for arterial pH outcomes: (A) overall pooled analysis; (B) sensitivity analysis.

Using the random effects model, we conducted a double‐armed meta‐analysis of 4 studies reporting on pH levels in DKA patients receiving bicarbonate therapy versus controls. Our analysis of 338 patients shows statistically insignificant changes in pH values post‐bicarbonate intervention compared to controls (mean difference = −0.02 [−0.13, 0.09], *p* = 0.7) (Figure 3A). The heterogeneity measure indicates high study variability (*I* ^2^ = 94%).

We conducted a leave‐one‐out test to address the high heterogeneity by excluding the studies by Gamba et al. and Ozturk et al. This analysis showed no statistically significant benefit of bicarbonate therapy (*p* = 0.18, *I* ^2^ = 0%) (Figure 3B).

**FIGURE 4:**
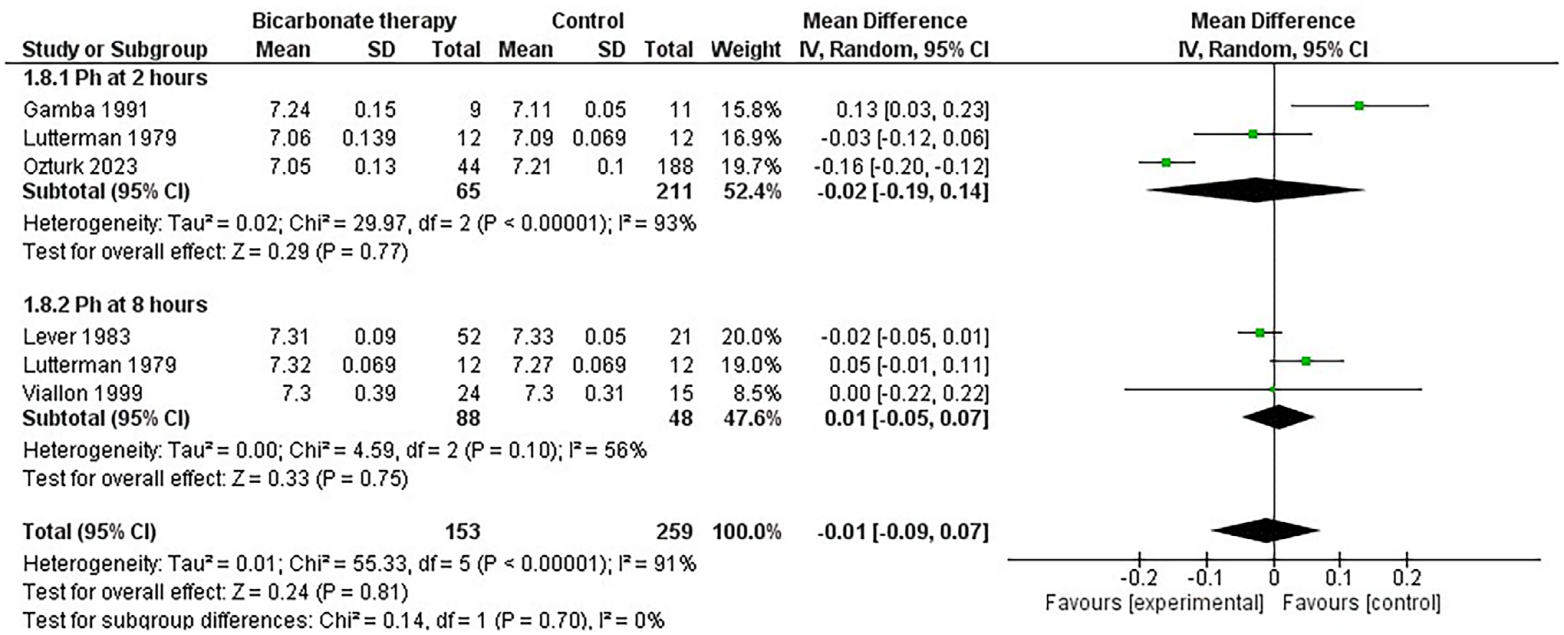
Subgroup analysis forest plot of arterial pH at different time points following bicarbonate administration (2 h vs. 8 h).

We further conducted a subgroup analysis of pH at 2 h and 8 h to address variability among the studies. The analysis showed no statistically significant difference in pH values between the bicarbonate therapy and control groups at 2 h (*p* = 0.77) and at 8 h (*p* = 0.75) (Figure 4).

The funnel plot did not show marked asymmetry, suggesting limited evidence of publication bias (File S1, Figure S1).

### 3.4 Duration of Hospital Stay

**FIGURE 5:**
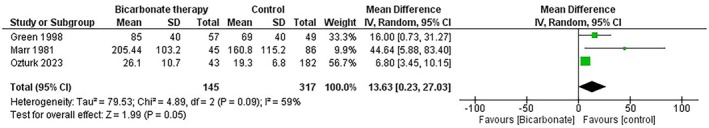
Forest plot comparing the length of hospital stay between the bicarbonate therapy and control groups (mean difference).

Using a random effects model, a meta‐analysis was conducted to evaluate the effect of bicarbonate therapy on the duration of hospital stay in diabetic ketoacidosis (DKA) patients. Three studies, comprising a total of 462 participants, were included in the analysis. The mean difference (MD) between the bicarbonate therapy and control groups was 13.63 h (95% CI [0.23, 27.03], *p* = 0.05, *I* ^2^ = 59%), indicating a marginally significant increase in hospital stay duration in the bicarbonate group compared to controls (Figure 5).

Among the studies, Ozturk et al. reported a smaller mean difference with less variability, while Green et al. and Marr et al. demonstrated wider confidence intervals, contributing to the overall heterogeneity.

### 3.5 Time to Resolution of Acidosis

**FIGURE 6:**
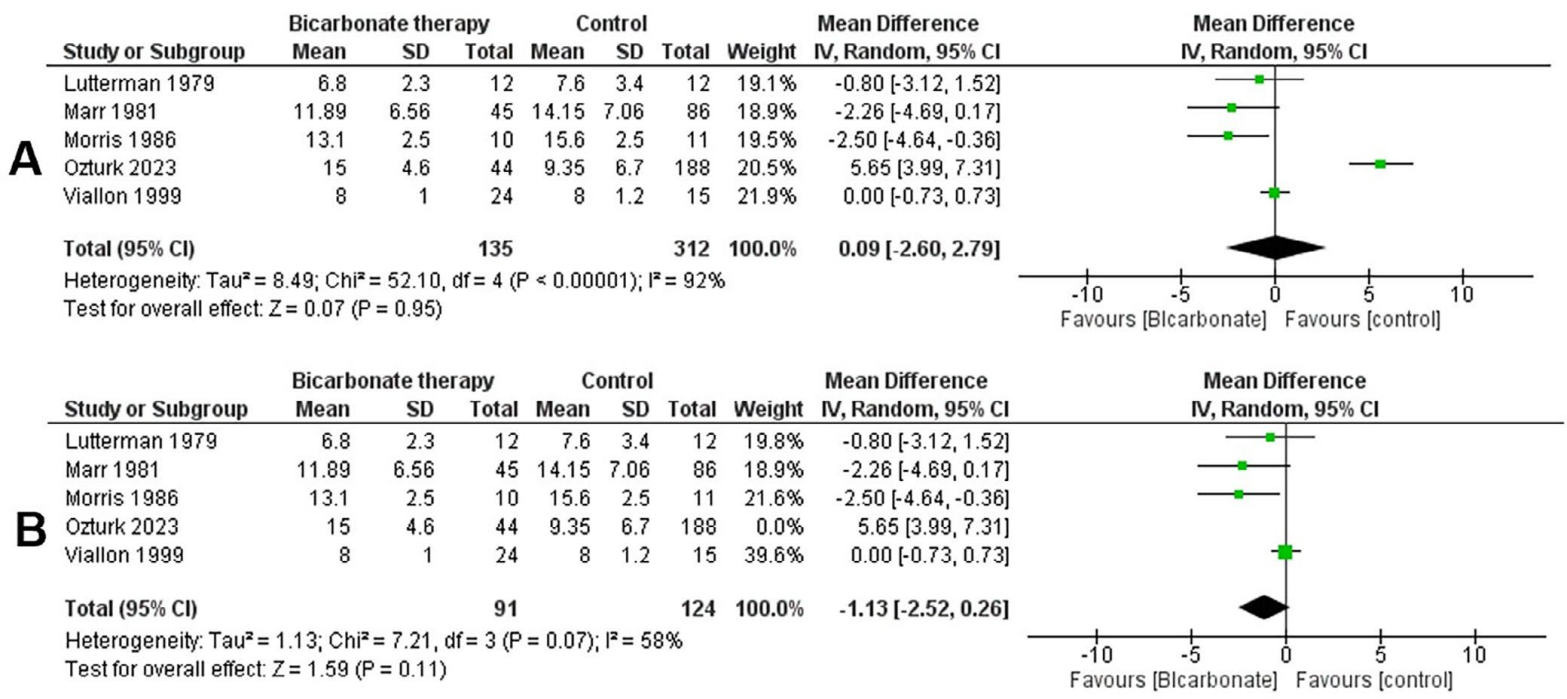
Forest plots comparing bicarbonate therapy versus control for Time to Resolution of Acidosis: (A) overall pooled analysis; (B) sensitivity analysis.

A meta‐analysis was conducted to assess the impact of bicarbonate therapy on the time to resolution of acidosis in diabetic ketoacidosis (DKA) patients. Five studies, including 447 participants, were analysed. The initial analysis showed a mean difference (MD) of 0.09 h (95% CI [−2.6, 2.79], *p* = 0.95), indicating no statistically significant difference between bicarbonate therapy and control groups. Heterogeneity was high (*I* ^2^ = 92%), suggesting high variability among the included studies (Figure 6A).

To address the high heterogeneity, a sensitivity analysis was performed by excluding the study by Ozturk et al. Exclusion of this study reduced heterogeneity (*I* ^2^ = 58%) and yet demonstrated no statistically significant reduction in the time to resolution of acidosis with bicarbonate therapy (MD = −1.13 h, 95% CI [−2.52, −0.26], *p* = 0.11) (Figure 6B).

The corresponding funnel plot (File S1, Figure S2) did not reveal clear asymmetry, although the small number of studies limits the reliability of publication‐bias assessment.

### 3.6 K+ Levels

**FIGURE 7:**
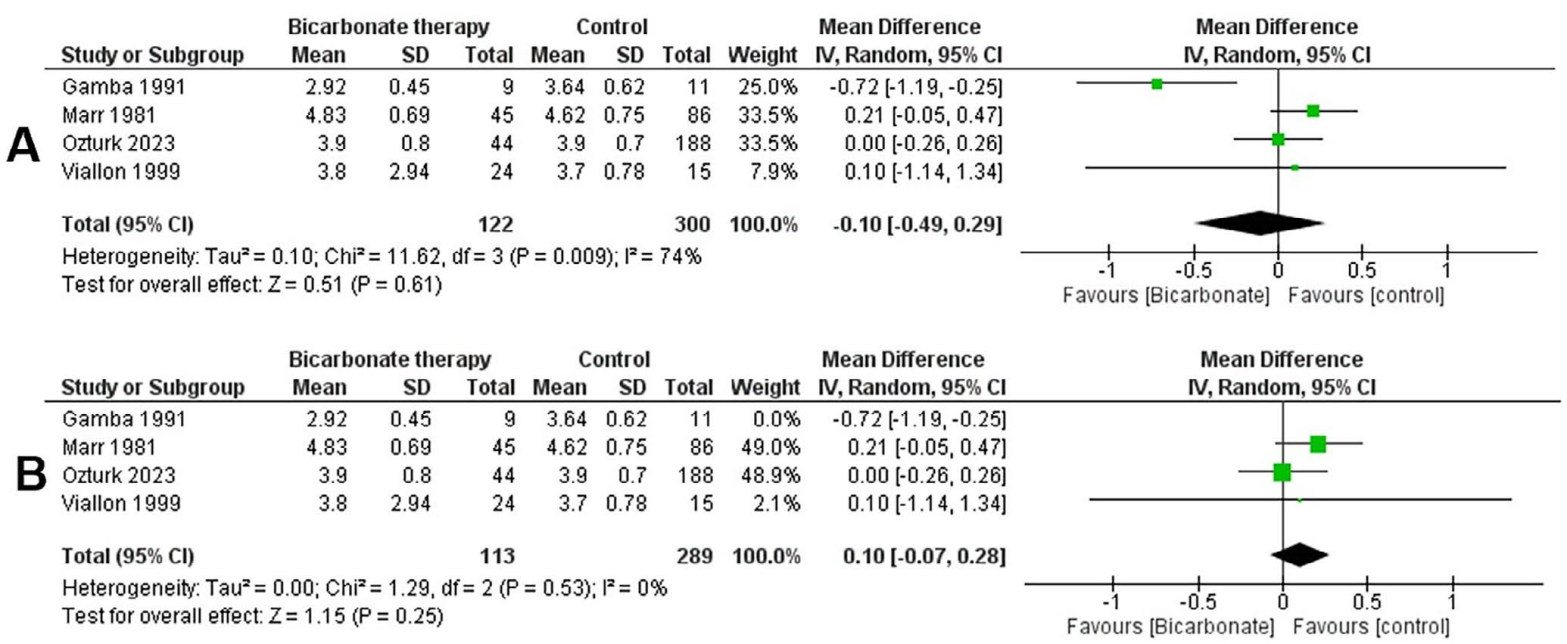
Forest plots comparing serum potassium levels between bicarbonate therapy and control groups: (A) overall analysis; (B) sensitivity analysis.

Using the random effects model, we performed a meta‐analysis of 4 studies evaluating potassium levels in DKA patients receiving bicarbonate therapy versus controls. A total of 422 patients were included. The analysis showed no statistically significant difference in potassium levels between the bicarbonate therapy and control groups (mean difference = −0.10 [−0.49, 0.29], *p* = 0.61). Heterogeneity among studies was high (*I* ^2^ = 74%) (Figure 7A).

To address heterogeneity, a leave‐one‐out sensitivity analysis was conducted. Excluding the study by Gamba et al. resulted in a mean difference favouring the control group, but the overall effect remained statistically insignificant (*p* = 0.32, *I* ^2^ = 22%) (Figure 7B).

The corresponding funnel plot (File S1 and Figure S3) did not demonstrate clear evidence of asymmetry, though the small number of studies limits meaningful interpretation.

### 3.7 HCO_3_ Levels

**FIGURE 8:**
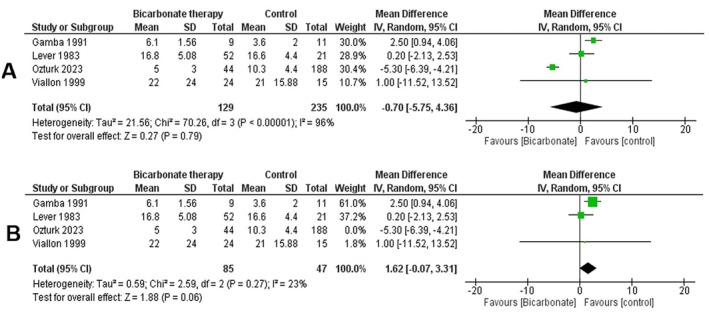
Forest plots comparing serum bicarbonate levels between the bicarbonate therapy and control groups: (A) overall analysis; (B) sensitivity analysis.

Using the random effects model, we conducted a double‐armed meta‐analysis of three studies reporting on HCO_3_ levels in diabetic ketoacidosis (DKA) patients receiving bicarbonate therapy versus controls. The analysis of 364 patients indicated statistically insignificant changes in HCO_3_ levels post‐bicarbonate intervention compared to controls (mean difference = −0.90 [−6.31, 4.51], *p* = 0.74, *I* ^2^ = 97%) (Figure 8A).

To address the high heterogeneity, a leave‐one‐out analysis was conducted. Excluding the study by Ozturk et al. reduced the heterogeneity (*I* ^2^ = 23%), with the mean difference favouring control group with no statistical significance (1.62 [−0.71, 3.75], *p* = 0.06) (Figure 8B).

The funnel plot for this outcome (File S1, Figure S4) showed no clear pattern of asymmetry, although the small number of studies limits the reliability of publication bias assessment.

### 3.8 Adverse Event: Hypoglycemia

A meta‐analysis was conducted using a fixed‐effects model to evaluate the incidence of hypoglycemia in patients with diabetic ketoacidosis (DKA) receiving bicarbonate therapy compared to a control group. Three studies, encompassing 118 participants (bicarbonate group: 74; control group: 44), were included in the analysis.

**FIGURE 9:**
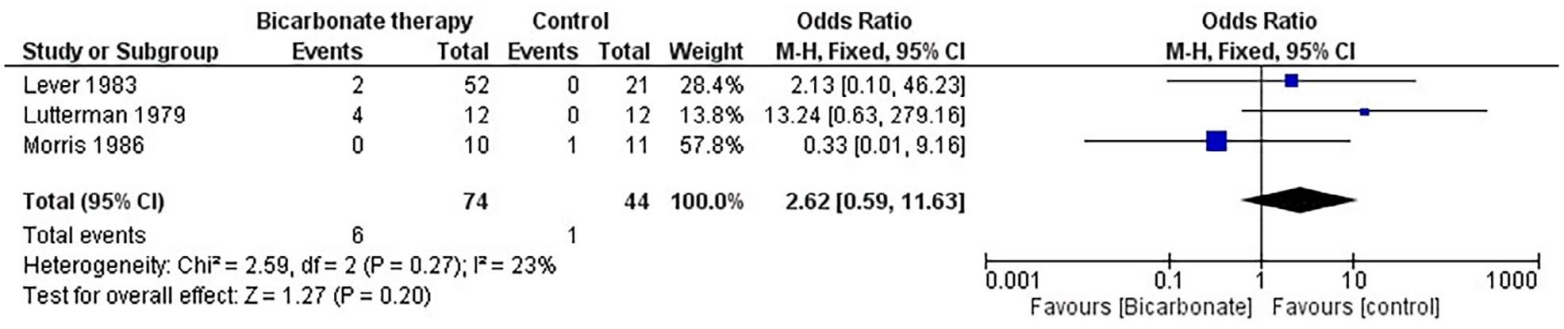
Forest plot comparing hypoglycemia outcomes between bicarbonate therapy and control groups (odds ratio).

The odds ratio (OR) for hypoglycemia in the bicarbonate therapy group compared to the control was 2.62 (95% CI [0.59, 11.63]; *p* = 0.20, *I* ^2^ = 23%), indicating no statistically significant difference between the groups. These results suggest that bicarbonate therapy in DKA does not significantly alter the risk of hypoglycemia compared to controls (Figure 9).

### 3.9 Glucose Levels

**FIGURE 10:**
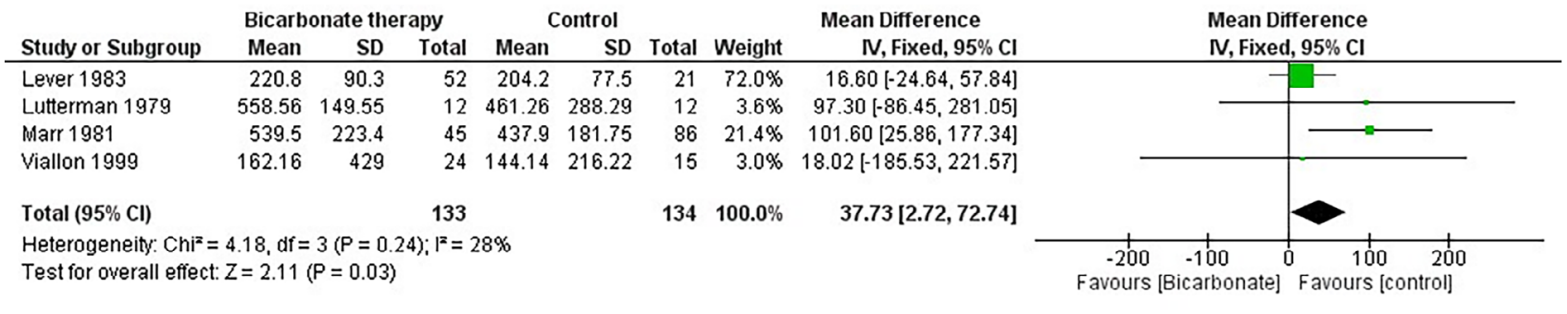
Forest plot comparing serum glucose levels between the bicarbonate therapy and control groups.

We conducted a meta‐analysis to evaluate the effect of bicarbonate therapy on glucose levels in patients with diabetic ketoacidosis (DKA). Four studies, including 267 participants, were analysed using a random‐effects model. The pooled analysis showed a mean difference (MD) of 37.73 mg/dL (95% CI [2.72, 72.74], *p* = 0.03), suggesting a statistically significant increase in glucose levels with bicarbonate therapy compared to the control group and the overall low heterogeneity supports the reliability of the pooled effect (*I* ^2^ = 28%) (Figure 10).

Lever et al. contributed most to the analysis (53.0%) and reported a mean difference of 16.60 mg/dL (95% CI [−24.64, 57.84]), which was not statistically significant. Marr et al. showed the largest mean difference of 101.60 mg/dL (95% CI [25.86, 177.34]), suggesting a significant increase in glucose levels with bicarbonate therapy.

The funnel plot for this outcome (File S1, Figure S5) appeared largely symmetric, although the limited number of studies restricts the strength of conclusions regarding publication bias.

## 4 Discussion

Our meta‐analysis demonstrates that bicarbonate therapy offers no significant advantage over standard care in DKA. We found no meaningful improvement in pH, time to resolution of acidosis, potassium or bicarbonate levels or risk of hypoglycemia, while observing signals of potential harm, including longer hospital stays and higher glucose levels in the bicarbonate group. These results are broadly consistent with the systematic review by Chua et al., which reported only transient early improvements in acidosis without sustained biochemical or clinical benefit and suggested a possible association with prolonged hospitalisation, particularly in paediatric DKA [36].

In light of these converging findings, it is essential to interpret our results within the context of current treatment guidelines, which generally discourage the routine use of bicarbonate in DKA. The American Diabetes Association (ADA) recommends bicarbonate only in cases of severe acidosis (pH < 6.9), as routine use is not supported by evidence and may not improve outcomes. Potential risks include delayed ketone clearance and electrolyte derangements [37]. Similarly, the International Society for Paediatric and Adolescent Diabetes (ISPAD) advises against routine use, citing a lack of clear benefit and potential for prolonged DKA resolution [38]. The European Society for Paediatric Endocrinology (ESPE) likewise cautions against routine bicarbonate therapy due to associated shifts in electrolytes and risk of complications [39]. Our findings align closely with these guidelines, particularly regarding the lack of improvement in acidosis parameters and the indication for a prolonged hospital stay.

Although DKA occurs across the lifespan, the physiological response to acidemia and the risks associated with its treatment differ substantially between adults and children. None of the studies included in our review provided age‐stratified outcome data, which prevented formal subgroup analyses. As a result, our findings should be interpreted as applying broadly to DKA rather than to specific age groups. Nevertheless, existing guidelines and observational evidence highlight important age‐related differences in the safety and potential role of bicarbonate therapy.

Guidelines consistently emphasise that bicarbonate therapy should be reserved for extreme acidemia rather than used routinely. In paediatric DKA, bolus bicarbonate administration has been associated with an increased risk of cerebral edema, and continuous bicarbonate infusions are generally considered only in highly selected situations such as prolonged non–anion gap metabolic acidosis [40]. Conversely, in adults, sodium bicarbonate may have a limited role in cases of profound, refractory acidemia accompanied by hemodynamic instability or life‐threatening hyperkalemia, but its use remains narrowly restricted to acute metabolic derangements rather than standard DKA management [41].

Although neurological complications—particularly cerebral edema—represent the most serious potential adverse effects of bicarbonate therapy, the studies included in our review did not report neurological outcomes, precluding a pooled meta‐analysis. Nonetheless, this complication warrants explicit discussion given its clinical significance. In a large paediatric case–control study of 6977 DKA episodes, Glaser et al. identified bicarbonate administration as the only treatment factor significantly associated with cerebral edema after adjustment for disease severity [38]. Although cerebral edema is rare in adults, isolated case reports—including a recent fatal event in a young adult treated with high‐dose bicarbonate—demonstrate that the risk may not be negligible [42]. Taken together, these findings underscore a biologically plausible and clinically meaningful safety concern, particularly in paediatric DKA, and reinforce current guideline recommendations against the routine use of bicarbonate therapy.

Heterogeneity was consistently high across several outcomes (pH, HCO_3_^−^, potassium and time to resolution of acidosis). This likely reflects variations in: (1) age groups (paediatric vs. adult populations), (2) treatment practices across different decades, (3) thresholds for bicarbonate administration, (4) dosing strategies and (5) baseline severity of acidosis. These factors limit the comparability of included studies and contribute to the wide variance in effect estimates. Sensitivity analysis reduced heterogeneity in several outcomes, but overall conclusions remained unchanged, reinforcing the robustness of the primary findings.

The cumulative evidence from this review—when examined alongside major diabetes guidelines—supports a consistent conclusion: bicarbonate therapy provides no clinically meaningful benefit in routine DKA management and may expose patients, particularly children, to unnecessary risks. Small signals suggesting increased hospital stay and worsened glycemic control further reinforce caution.

Given the absence of improvement in key clinical outcomes, and in light of potential adverse effects, bicarbonate therapy should remain reserved for the narrow subset of patients with extreme acidemia (pH < 6.9) or refractory metabolic instability, where its use may be justified on physiological grounds.

Several gaps in the literature warrant further investigation. Multicenter randomised controlled trials with standardised treatment protocols are needed to strengthen the evidence base. Studies evaluating newer buffering agents, such as Tris(hydroxymethyl)aminomethane (THAM), remain limited [43]. Additionally, comparative analyses in low‐resource settings may help elucidate context‐specific risks and benefits where DKA severity and treatment resources differ from high‐income settings. Importantly, none of the available studies stratified outcomes by diabetes type, preventing assessment of whether bicarbonate therapy may have differential effects in type 1 versus type 2 diabetes. Future research should therefore incorporate diabetes‐type–specific analyses to clarify potential phenotype‐specific risks or therapeutic responses.

## 5 Limitations

Several limitations should be acknowledged. The inclusion of older studies with outdated treatment paradigms may limit the applicability of our results to modern clinical practice, although this was necessary due to the scarcity of recent studies with extractable data. Additionally, some outcomes were derived from very small sample sizes, most notably hypoglycemia, which was assessed across only three studies with a combined total of 118 participants. This limited sample size reduces statistical power and restricts the confidence with which these findings can be interpreted.

A major limitation of this meta‐analysis is the significant clinical heterogeneity among the included studies, stemming from variations in patient age, baseline severity of acidosis, comorbidities, fluid and insulin protocols and bicarbonate dosing strategies. These discrepancies compromise comparability and limit the generalizability of conclusions.

Another key limitation is the lack of data on neurological complications such as cerebral edema, which prevented quantitative pooling of these outcomes. This restricts the clinical relevance of the findings, particularly for paediatric DKA.

Furthermore, the meta‐analysis pooled paediatric and adult populations without age‐stratified subgroup analyses, which may obscure age‐specific risks—especially given that cerebral edema risk is concentrated in children.

Finally, although funnel plots were generated, the limited number of included studies for each outcome reduces the reliability of publication‐bias assessment, and formal statistical tests such as Egger's regression could not be performed.

## 6 Conclusion

Our systematic review and meta‐analysis demonstrate that bicarbonate therapy offers no meaningful clinical advantage in the routine management of diabetic ketoacidosis (DKA). Across all evaluated outcomes—including pH correction, time to acidosis resolution, potassium levels, glucose levels and hospital stay—bicarbonate therapy did not improve patient outcomes and, in some cases, was associated with potential harms such as exacerbation of hyperglycemia.

Consistent with current ADA and ISPAD guidelines, bicarbonate therapy should not be used routinely in DKA. Its use should be strictly limited to rare situations of severe or refractory acidosis (pH < 6.9), where potential benefits may outweigh risks. Given the limited number and quality of available studies, further well‐designed trials are needed to clarify the role of bicarbonate in extreme acidemia and to evaluate alternative buffering strategies.

## Author Contributions

Abu Omayer: conceptualization, data curation, writing, analysis and reviewing. KC Anil: conceptualization and data curation. Arsalan Sharif: data curation, writing and reviewing. Syed Mohammed Hassanul Hoque: data curation and writing. Hasan BaniHani: writing and reviewing. Lana Khaled: writing. Waseem Sajjad: reviewing. Moussa Nassar: reviewing and correspondence. Ahmad Holeihel: reviewing and supervision. All authors reviewed and approved the final version of the manuscript.

## Funding

The authors have nothing to report.

## Ethics Statement

The authors have nothing to report.

## Consent

The authors have nothing to report.

## Conflicts of Interest

The authors declare no conflicts of interest.

## Supporting information


**Data S1:** Electronic database search strategies (Google Scholar, PubMed, Cochrane) and Funnel plots (Figures S1–S5).

**Table S1:** PRISMA 2020 reporting checklist with manuscript page references.

## Data Availability

The data that support the findings of this study are available from the corresponding author upon reasonable request.
