# Supplementary material for: The Role of Bicarbonate Therapy in Diabetic Ketoacidosis: A Systematic Review and Meta‐Analysis
Source: Endocrinol Diabetes Metab. 2026 Mar 9;9(2):e70191. doi: 10.1002/edm2.70191 (PMC12968726; doi:10.1002/edm2.70191)
Supplement: Supplementary file 1 — Data S1: Electronic database search strategies (Google Scholar, PubMed, Cochrane) and Funnel plots (Figures S1–S5). [file EDM2-9-e70191-s001.docx]

**GOOGLE SCHOLAR SEARCH STRATEGY:**

"diabetic ketoacidosis"|"Diabetic Ketoacidosis"|"diabetic ketosis"|DKA|"diabetic acidosis"|ketoacidosis bicarbonate|Bicarbonates|"sodium bicarbonate"|"Buffering Agents"|"hydrogen carbonates"|"sodium hydrogen carbonate"|"bicarbonate therapy"|"alkalinizing agents" treatment|treatments|Therapeutics|management|intervention "Randomized Controlled Trial"|"RCT"|"Clinical Trial"|"Prospective Study"|"Cohort Study"|"Cross-Sectional Study"|"Case Series"|"observational study"|"retrospective study"|"comparative study"

**900+results**

**PUBMED SEARCH STRATEGY:**

("diabetic ketoacidosis" OR "Diabetic Ketoacidosis"[Mesh] OR "diabetic ketosis" OR DKA OR "diabetic acidosis" OR ketoacidosis) AND (bicarbonate OR "Bicarbonates"[Mesh] OR "sodium bicarbonate" OR "Buffering Agents"[Mesh] OR "hydrogen carbonates" OR "sodium hydrogen carbonate" OR "bicarbonate therapy" OR "alkalinizing agents") AND (treatment OR treatments OR "Therapeutics"[Mesh] OR management OR intervention) AND ("Randomized Controlled Trial"[Publication Type] OR "Randomized Controlled Trials as Topic"[Mesh] OR "Clinical Trial"[Publication Type] OR "Clinical Trials as Topic"[Mesh] OR "Prospective Studies"[Mesh] OR "Cohort Studies"[Mesh] OR "Cross-Sectional Studies"[Mesh] OR "Case Series"[Publication Type] OR "observational study" OR "retrospective study" OR "comparative study")

**251 RESULTS**

**Cochrane**

**
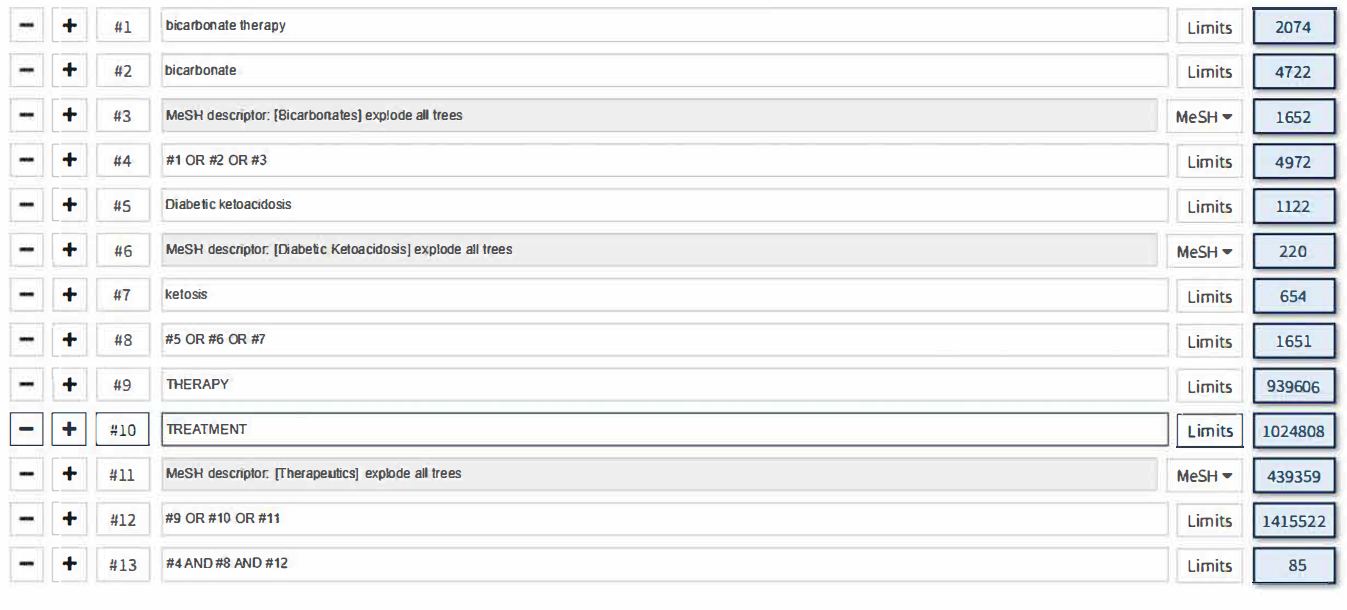
**

**Clinical trials**

**
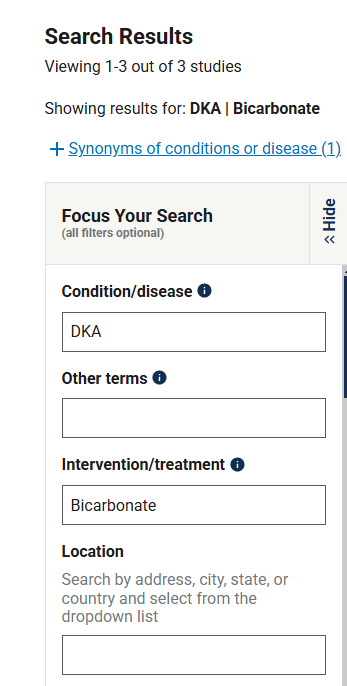
**


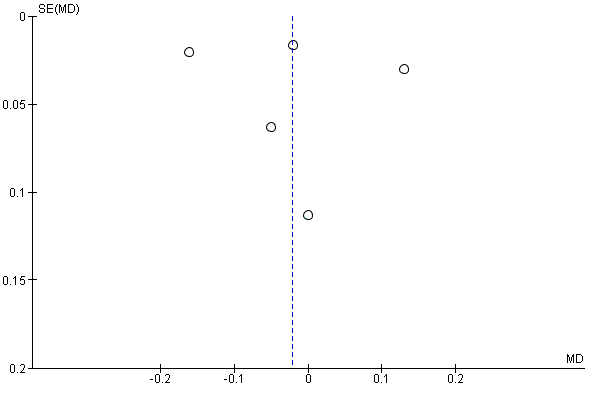


**Figure S1:** Funnel plot for pH outcome.


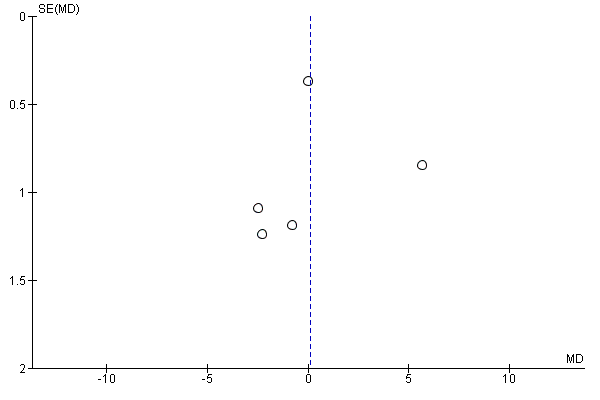


**Figure S2:** Funnel plot for time to resolution of acidosis.


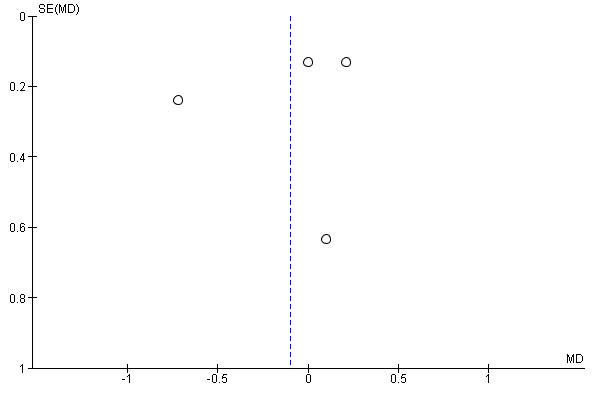


**Figure S3:** Funnel plot for potassium (K⁺) levels.


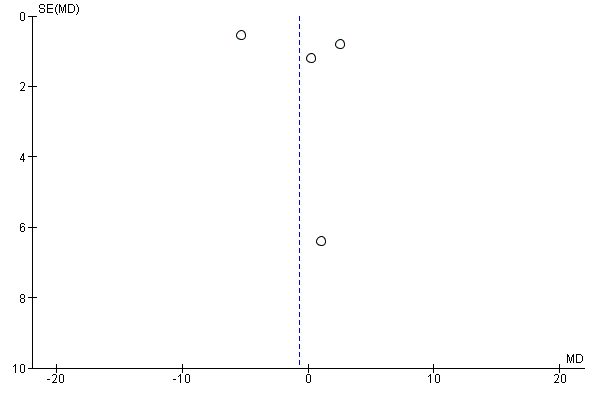


**Figure S4:** Funnel plot for bicarbonate (HCO₃⁻) levels.


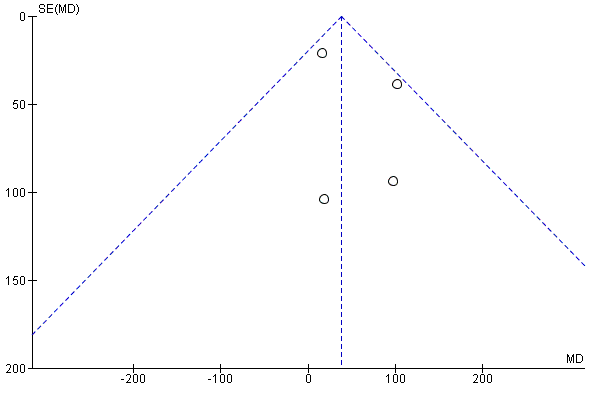


**Figure S5:** Funnel plot for glucose levels.
